# Radiation Dose of Contrast-Enhanced Mammography: A Two-Center Prospective Comparison

**DOI:** 10.3390/cancers14071774

**Published:** 2022-03-31

**Authors:** Gisella Gennaro, Andrea Cozzi, Simone Schiaffino, Francesco Sardanelli, Francesca Caumo

**Affiliations:** 1 Unit of Breast Radiology, Veneto Institute of Oncology (IOV) IRCCS, Via Gattamelata 64, 35128 Padua, Italy; francesca.caumo@iov.veneto.it; 2 Department of Biomedical Sciences for Health, Università degli Studi di Milano, Via Mangiagalli 31, 20133 Milano, Italy; andrea.cozzi1@unimi.it (A.C.); francesco.sardanelli@unimi.it (F.S.); 3 Unit of Radiology, IRCCS Policlinico San Donato, Via Morandi 30, 20097 San Donato Milanese, Italy; simone.schiaffino@grupposandonato.it

**Keywords:** breast cancer, contrast-enhanced mammography, radiation dose

## Abstract

**Simple Summary:**

Contrast-enhanced mammography (CEM) is a dual-energy technique where low- and high-energy images are acquired for each mammographic view after contrast agent administration, and are then recombined to enhance potential contrast uptake. As CEM is increasingly used for both screening and diagnostic applications in breast imaging, but its associated radiation dose has been investigated only by single-center studies, we aimed to evaluate the CEM per-patient radiation dose on a large population in a bicentric setting, pooling data from two prospective studies employing the same model of mammography units. The CEM radiation dose showed a 6.2% difference between the two centers, mainly attributable to the study populations’ characteristics and to manufacturing differences between the two systems. The CEM dose was about 30% higher than that of standard digital mammography. Such an increment was close to the dose increase reported for digital breast tomosynthesis, which is already used in both screening and clinical settings. Thus, considering the extensively demonstrated diagnostic gain granted by CEM over these non-contrast-enhanced techniques, radiation dose concerns should not hinder ever-wider clinical implementations of CEM.

**Abstract:**

The radiation dose associated with contrast-enhanced mammography (CEM) has been investigated only by single-center studies. In this retrospective study, we aimed to compare the radiation dose between two centers performing CEM within two prospective studies, using the same type of equipment. The CEM mean glandular dose (MGD) was computed for low energy (LE) and high energy (HE) images and their sum was calculated for each view. MGD and related parameters (entrance dose, breast thickness, compression, and density) were compared between the two centers using the Mann–Whitney test. Finally, per-patient MGD was calculated by pooling the two datasets and determining the contribution of LE and HE images. A total of 348 CEM examinations were analyzed (228 from Center 1 and 120 from Center 2). The median total MGD per view was 2.33 mGy (interquartile range 2.19–2.51 mGy) at Center 1 and 2.46 mGy (interquartile range 2.32–2.70 mGy) at Center 2, with a 0.15 mGy median difference (*p* < 0.001) equal to 6.2%. LE-images contributed between 64% and 77% to the total patient dose in CEM, with the remaining 23–36% being associated with HE images. The mean radiation dose for a two-view bilateral CEM exam was 4.90 mGy, about 30% higher than for digital mammography.

## 1. Introduction

Contrast-enhanced mammography (CEM) is a dual-energy technique in which two images for each mammographic view are acquired after the administration of an iodinated contrast agent during a single breast compression [1,2,3]. From a technical point of view, CEM images are obtained by separating the two X-ray spectra, so that the first is kept below the iodine absorption peak at 33.2 keV (low-energy [LE] image) and the second is pushed above the 33.2 keV absorption peak (high-energy [HE] image) [3]. CEM is interpreted by considering both the LE image—equivalent to a standard digital mammography (DM) image [4]—and a dual-energy image obtained from the recombination of LE and HE images, showing contrast enhancement of hypervascularized lesions and of the parenchymal background [3,5]. Contrast enhancement reveals the neoangiogenesis and the expansion of the extracellular volume associated with breast cancer and other breast lesions, providing functional information combined with the high-resolution morphological information of LE images [6,7]. Thanks to its twofold diagnostic profile, CEM performance has been reported as higher than DM or digital breast tomosynthesis (DBT) and as comparable to that of breast contrast-enhanced MRI [7,8].

As the CEM radiation dose is the sum of the doses associated with LE and HE images, and LE images are substantially DM images, CEM radiation dose is expected to be higher than that of DM. The few studies comparing CEM, DM, and DBT doses confirmed that CEM delivers a radiation dose higher than DM, and comparable to the one of DBT [9,10,11].

While concerns about risks associated with the exposure to ionizing radiation are limited and outweighed by potential benefits when an imaging technique is used in symptomatic patients or for characterizing suspicious findings, cancer staging, or neoadjuvant therapy evaluation [1,6,7], dose assessment becomes far more important if an imaging technique (in this case, CEM) is used to image healthy subjects, as occurs in screening populations [12,13,14,15,16,17]. Thus, assessment of the CEM radiation dose is crucial for defining its clinical application field.

This study aimed to retrospectively compare CEM radiation doses in two populations from two prospective studies where CEM was acquired with the same type of mammography unit and with the same acquisition protocol. In one study, CEM serves as a screening tool for women at increased breast cancer risk, while in the other, CEM is used in the work up of suspicious findings detected at screening mammography.

## 2. Materials and Methods

### 2.1. Study Population

**Figure 1:**
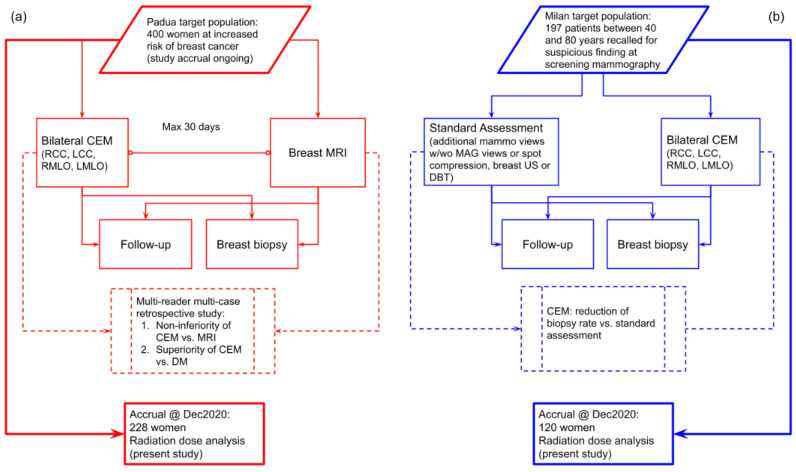
(**a**) Flowchart of the prospective study ongoing at Center 1 comparing CEM with breast MRI in a population of women at increased risk of breast cancer. (**b**) Flowchart of the prospective study at Center 2 using CEM as a work-up tool for suspicious findings detected at screening mammography.

This observational study is a pooled analysis of data from two prospective studies using CEM in different settings, which had in common the secondary endpoint of evaluating radiation dose. The flowcharts of the two studies are depicted in Figure 1.

The study at Center 1 (Veneto Institute of Oncology (IOV)—IRCCS, Padua, Italy), approved by the Institutional Ethics Committee on 22 December 2017 (protocol code #2017/92), is enrolling women at increased risk for breast cancer (assessed using the Tyrer–Cuzick model) with the aims of testing CEM non-inferiority compared to breast MRI and CEM superiority over DM through a multi-reader multi-case ROC analysis. The study at Center 2 (IRCCS Policlinico San Donato, San Donato Milanese, Italy), approved by the Institutional Ethics Committee on 10 May 2018 (protocol code CESM), enrolled women recalled from mammography screening who underwent CEM in addition to standard work-up (supplemental DM or DBT views, and/or breast ultrasound), aiming to evaluate the CEM potential for reducing the biopsy rate. In both studies, all enrolled patients signed informed consent. CEM examinations in both centers were performed using the same model of mammography unit (GE Senographe Pristina, General Electric Healthcare, Chicago, IL, USA), and the same clinical protocol: cranio-caudal (CC) views followed by the medio-lateral oblique (MLO) views, starting 2 min after the administration of a 1.5 mL/kg dose of an iodinated contrast agent (Iohexol 350 mgI/mL) with a 3.0 mL/s flow rate.

### 2.2. Technical Comparison of Contrast-Enhanced Mammography Units

The two CEM units were installed in May 2018 at Center 1 and in March 2018 at Center 2. As a preliminary step, a technical comparison between the CEM units located at Center 1 and Center 2 was performed. X-ray tube performance was compared measuring tube outputs and half value layers (HVLs) using a RaySafe X2 multimeter equipped with a MAM sensor (Unfors RaySafe AB, Billdal, Sweden). Three tube output and HVL measurements were acquired for the two pairs of X-ray spectra used by the automatic exposure control (AEC) for CEM acquisition. AEC and detector performance were compared between the two centers by measuring, separately for LE- and HE-images, the following parameters: (i) entrance dose (ED) values as a function of breast phantom thickness; (ii) contrast-to-noise ratio (CNR), as image quality index, as a function of breast phantom thickness; (iii) response function; and (iv) noise. AEC performance was evaluated by using breast phantoms of different thicknesses assembled by stacking semi-circular polymethyl-methacrylate (PMMA) slabs (from 20 to 70 mm thick, at 5 mm intervals), on top of which a thin (0.2 mm) aluminum square (15 × 15 mm^2^) was superimposed to produce image contrast. One image in the AEC mode was acquired for each phantom thickness and CEM unit. The ED was calculated by multiplying the tube output previously measured by the exposure (mAs) value selected by the AEC, adjusting the resulting value for the source-to-phantom-entrance distance. Then, the CNR, i.e., the absolute difference between the mean signal measured within the aluminum square and the mean signal measured in the PMMA background surrounding the aluminum square divided by the noise in the PMMA background [18], was measured from phantom images using ImageJ2 [19]. The detector performance was determined by exposing a standard PMMA phantom 45 mm thick in manual exposure mode, using the same X-ray spectra used by the AEC for this object thickness level, while selecting increasing exposure (mAs) values to cover the full clinical exposure range. Mean pixel value (MPV) and standard deviation (SD) in a region of interest (ROI) at about 5 cm from the chest wall were measured with ImageJ2, and were used as the signal and noise metrics, respectively. The response function was obtained by plotting the MPV as a function of ED, and interpolating the experimental data with a linear fit; R^2^ > 0.99 was considered indicative of a linear detector response. The noise components were subsequently derived from SD^2^ (variance) as a function of MPV, and by interpolating the experimental data with a second order polynomial fit, according to the noise model proposed by Bouwman et al. [20]; R^2^ > 0.99 was considered indicative of good model fitting.

All of the aforementioned measurements that required image analysis were performed using DICOM “For Processing” images.

Relative differences (i.e., the absolute difference divided by the mean value) between each physical variable measured for the two CEM units were used to assess the technical differences between the two systems. Relative differences below 5% were considered representative of the normal variability between systems.

### 2.3. Clinical Dose Comparison and Statistical Analysis

LE images (DICOM “For Processing” format) from Center 1 and Center 2 were processed by Volpara algorithm v.1.5.5.1 (Volpara Health Ltd., Wellington, New Zealand) to determine volumetric breast density and MGD associated with LE images [21], MGD values being adjusted for individual breast density. Other parameters used to calculate MGD were obtained from the image DICOM header, such as ED, compressed breast thickness, and HVL. MGD associated with the HE images was computed using ED, compressed breast thickness, and HVL recorded in the DICOM header, and the conversion factors published by Dance et al. [22,23]. The total MGD for each CEM mammographic view was obtained as the sum of LE-and HE-MGDs. Differences in breast thickness, compression force, volumetric breast density, LE- and HE-ED, exposure (mAs), and total MGD between Center 1 and Center 2 datasets were assessed with the Mann–Whitney U test.

Finally, pooling the two datasets together, per-patient total CEM MGD was calculated by summing MGDs from CC and MLO CEM views for each breast and averaging the two values obtained for the left and right breasts, together with the proportion of CEM dose associated with LE and HE images as a function of breast thickness.

Statistical analyses were performed with MedCalc (version 20.009, MedCalc Software Ltd., Ostend, Belgium), with *p* values < 0.05 indicating a statistically significant difference.

## 3. Results

### 3.1. Study Population

This analysis on CEM radiation dose included 228 women (451 CC and 455 MLO views) from Center 1 and 120 women (243 CC and 241 MLO views) from Center 2, for a total of 348 women and 1390 views. Women from Center 1 were enrolled between 1 March 2019 and 31 December 2020, while women from Center 2 were enrolled between 25 January 2019 and 21 February 2020. Mean age (± standard deviation) was significantly different in the two datasets: 51 ± 9 years for women enrolled at Center 1 and 59 ± 10 years for women enrolled at Center 2 (*p* < 0.001). The Center 1 dataset included 172/228 (75.4%) high-risk and 56/228 (24.6%) intermediate-risk women, while the Center 2 dataset included women with any breast cancer risk profile without any preliminary risk assessment. Breast density was also different between the two centers: 77.6% (177/228) of women enrolled by Center 1 had dense breasts (category *c* and *d* of the Breast Imaging Reporting and Data System classification), compared to 45.0% (54/120) of women from Center 2 (*p* < 0.001). The differences between the two datasets that constitute this study population are summarized in Table 1.

### 3.2. Technical Comparison of Contrast-Enhanced Mammography Units

Table 2 reports the tube output and HVL measurements for the two centers and related relative differences for the only two anode/filter/kV_p_ pairs used by the AEC in CEM mode: below 35 mm thickness the molybdenum (Mo) target material combined with a Mo filter—setting tube voltage at 26 kV_p_—was used for the LE-image, while the same Mo anode with copper (Cu) filtration—setting tube voltage at 49 kV_p_—was used for the HE-image; for object thickness equal or above 35 mm, the rhodium (Rh) target material was used in combination with a silver (Ag) filter at 34 kV_p_ for the LE-image and with a Rh/Cu filter at 49 kV_p_ for the HE-image.

**Figure 2:**
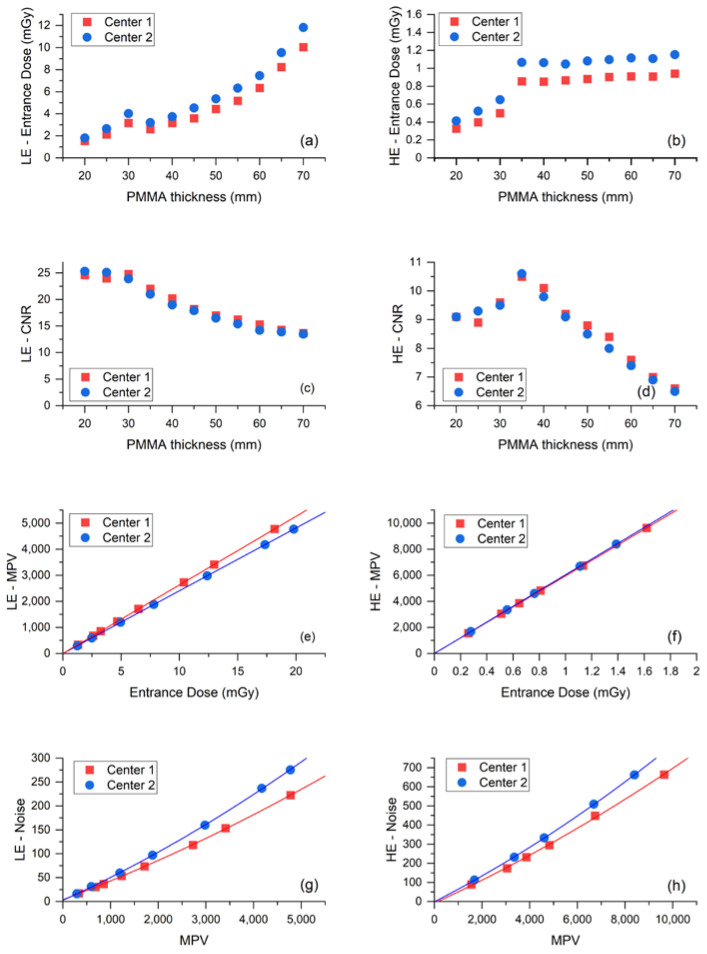
(**a**) ED used by the AEC to acquire the LE-images of PMMA phantoms as a function of phantom thickness. (**b**) ED used by the AEC to acquire the HE-images of PMMA phantoms as a function of phantom thickness. (**c**) Contrast-to-noise ratio (CNR) measured from the LE-images as a function of PMMA thickness. (**d**) CNR measured from the HE-images as a function of PMMA thickness. (**e**) Response function (MPV vs. ED) for LE-image acquisition. (**f**) Response function for HE-image acquisition. (**g**) Noise evaluation (SD^2^ vs. MPV) for LE-image acquisition. (**h**) Noise evaluation for HE-image acquisition.

The tube output was slightly higher at Center 1 than Center 2, while the opposite was true for the HVL of the LE X-ray beams. However, the relative differences were all below 5%, a value considered compatible with acceptable manufacturing differences and measurement uncertainty. Comparisons of all other measurements regarding AEC and detector performance are shown in Figure 2.

Plots in Figure 2a,b show that the Center 2 AEC systematically used an higher ED than Center 1 to expose the same test objects, for both LE- and HE-images. Conversely, Figure 2c,d indicate that the resulting CNR values produced by the two systems are substantially equivalent for both LE- and HE-images. Relative differences of ED and CNR for LE- and HE-images are listed in Table 3 for each phantom thickness and on the average.

**Figure 3:**
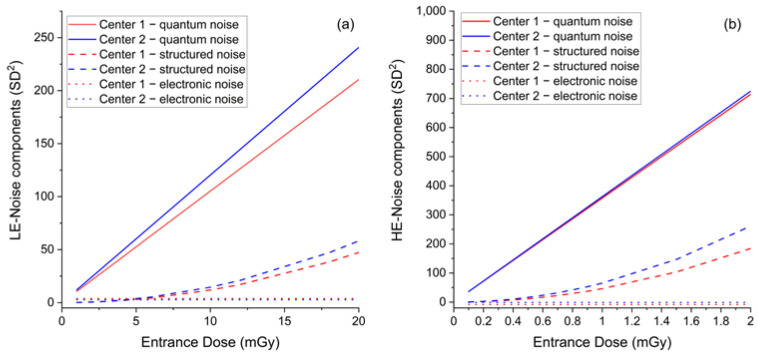
(**a**) Comparison between LE-noise components as a function of ED between the two centers. (**b**) Comparison between HE-noise components as a function of ED between the two centers.

The mean ED relative difference was 4.8% for LE-images and 5.5% for HE-images, while the mean CNR relative differences were 0.7% and 0.3%, respectively. The systematical increase of ED generated by the AEC in Center 2 compared to Center 1 needed to achieve the same image quality (CNR), which suggests the existence of some differences between the detectors of the two mammography units. Indeed, the response function and noise analyses—depicted in panel e to panel h of Figure 2—show that the image detector used by the CEM system in Center 2 was slightly less sensitive than the detector in Center 1 in LE- mode, and noisier in both LE- and HE- modes. As expected, the response functions were linear for both centers, in both LE- and HE- acquisition mode (LE—Center 1: MPV = 1.30 + 263.00 × ED, R^2^ = 0.99999; LE—Center 2: MPV = −0.13 + 240.80 × ED, R^2^ = 1; HE—Center 1: MPV = 12.86 + 5944.08 × ED, R^2^ = 1; HE—Center 2: MPV = 0.58 + 6039.82 × ED, R^2^ = 0.99998). The relative difference between the two detector sensitivities (obtained from the relative difference between the linear fit slopes) was 8.8% for LE-images and 1.6% for HE-images. Noise analysis confirmed that for both equipment, the principal noise component was quantum noise, followed by some structured noise, with minor electronic noise for both LE- and HE-images (LE—Center 1: noise = 3.90 + 0.04 × MPV + 1.71 × 10^−6^ × MPV^2^, R^2^ = 0.99988; LE—Center 2: noise = 2.71 + 0.05 × MPV + 2.51 × 10^−6^ × MPV^2^, R^2^ = 0.99991; HE—Center 1: noise = −8.23 + 0.06 × MPV + 1.30 × 10^−6^ × MPV^2^, R^2^ = 0.99856; HE—Center 2: noise = −1.16 + 0.06 × MPV + 1.79 × 10^−6^ × MPV^2^, R^2^ = 0.99991). Plots of LE- and HE-noise components as a function of ED are depicted in Figure 3, showing the difference between the two centers for each noise component. The relative difference between the prevalent quantum noise components was 22.2% in the LE-mode, while there was no difference in the HE-mode.

### 3.3. Clinical Dose Comparison

**Figure 4:**
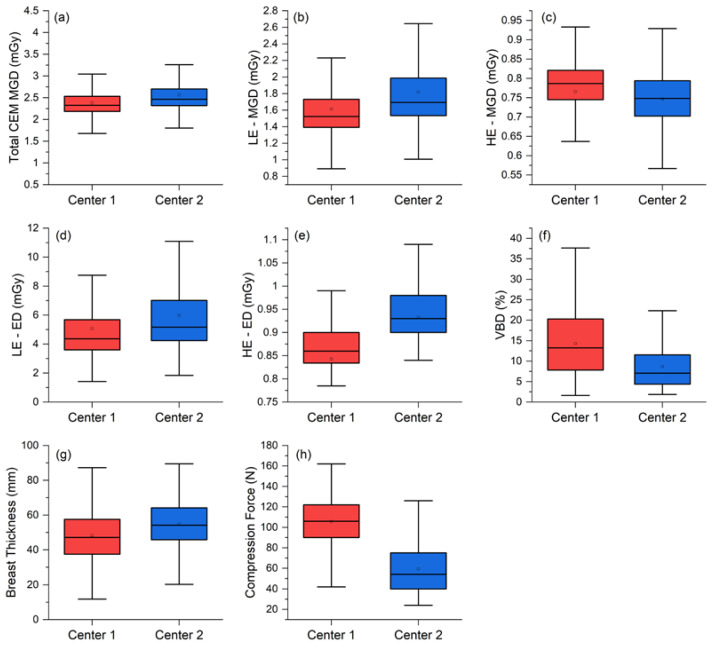
Per-view comparison between the two centers for the (**a**) total MGDs, (**b**) LE-MGD, (**c**) HE-MGD, (**d**) LE-ED, (**e**) HE-ED, (**f**) volumetric breast density (VBD), (**g**) breast thickness, and (**h**) compression force.

Figure 4 and Table 4 detail inter-center per-view comparison of the total CEM MGD, LE-MGD, HE-MGD, and LE- and HE-ED, volumetric breast density, compressed breast thickness, and compression force distributions.

The median total MGD per view was 2.33 mGy (interquartile range (IQR) 2.19–2.51 mGy) at Center 1 and 2.46 mGy (IQR 2.32–2.70 mGy) at Center 2, with a statistically significant 0.15 mGy median difference (*p* < 0.001). Analyzing the MGD components, LE-MGD was lower at Center 1 (Center 1: median 1.52 mGy, IQR 1.39–1.73 mGy; Center 2: median 1.69 mGy, IQR 1.54–1.99 mGy; *p* < 0.001), while HE-MGD was lower at Center 2 (Center 1: median 0.79 mGy, IQR 0.75–0.82 mGy; Center 2: median 0.75 mGy; IQR 0.70–0.79 mGy; *p* < 0.001). ED components (LE and HE) were both lower at Center 1 (LE-ED: median 4.37 mGy, IQR 3.60–5.68 mGy; HE-ED: median 0.86 mGy, IQR 0.83–0.90 mGy) than at Center 2 (LE-ED: median 5.18 mGy, IQR 4.24–7.01 mGy; HE-ED: median 0.93 mGy, IQR 0.90–0.98 mGy), and both differences were statistically significant (*p* < 0.001).

The median breast thickness in Center 2 was significantly greater than in Center 1 (Center 1: median 47.2 mm, IQR 37.5–57.6 mm: Center 2: median 54.2 mm, IQR 45.7–64.2 mm; *p* < 0.001), very likely associated with the significantly lower compression force applied by Center 2 (Center 1: median 106 N, IQR 90–122 N; *p* < 0.001). Finally, the volumetric breast density was significantly higher in Center 1 (Center 1: median 13.2%, IQR 7.8–20.3%; Center 2: median 7.1%, IQR 4.4–11.6%, *p* < 0.001).

**Figure 5:**
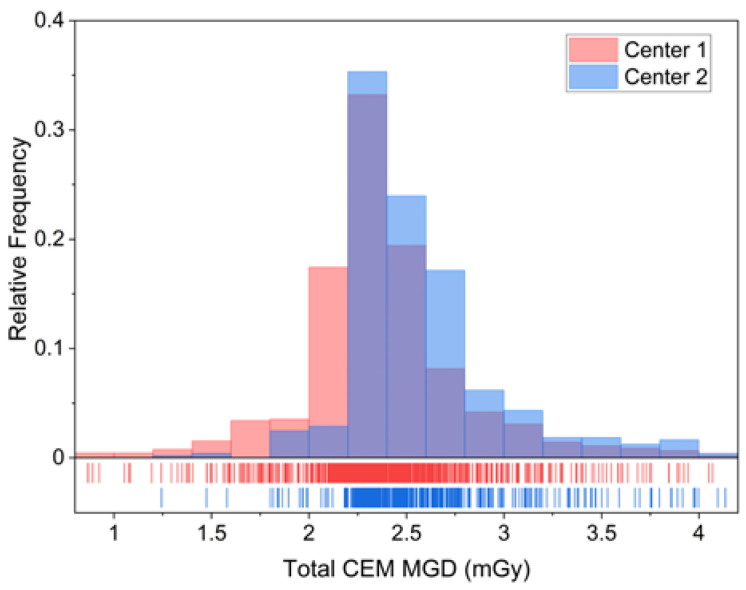
Total CEM MGD histograms for Center 1 (red) and Center 2 (blue). The relative frequency instead of counts was used as the y-axis in order to cope with the difference in size between the two datasets. The degree of overlap between the two distributions is shown in the darker central part of the plot. The rug at the bottom is composed by the projections of data points on the CEM MGD continuous axis by means of thin lines, better showing the slight dose increase by the CEM system at Center 2.

In Figure 5, the two per-view CEM MGD histograms are represented together with a “rug” density plot, showing the degree of dose overlap and the amount of MGD shift between the two centers.

Looking at the result of the superimposition of the two CEM MGD histograms in Figure 5, it can be noticed how the two MGD distributions representing the CEM dose at Center 1 and Center 2 clearly seem to belong to the same dose distribution: the minor and clinically negligible shift of the dose distribution at Center 2 is attributable to technical differences between the equipment, differences between the study populations, and differences in breast compression between the two centers. Considering this observed substantial overlap of the MGD distributions, we proceeded with data pooling to obtain the overall patient dose estimation. CEM per-patient MGD was computed as the sum of CEM MGD due to the CC and MLO view for each breast, averaged across the two breasts.

**Figure 6:**
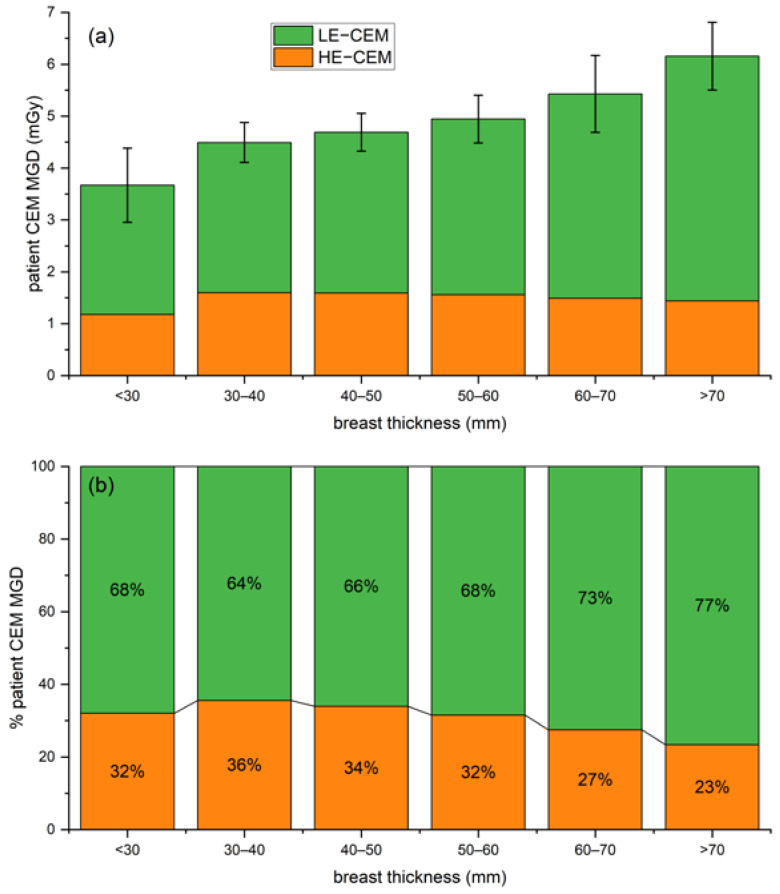
(**a**) Stacked column plot of overall patient MGD associated with CEM (LE in orange and HE in green) for increasing breast thickness ranges. (**b**) Normalized stacked plot showing the percentage of patient dose due to LE and HE images for increasing breast thickness ranges.

Figure 6a shows that the mean patient MGD progressively increases with breast thickness for LE acquisitions (from 2.49 mGy for less than 30 mm breast thickness to 4.72 mGy for breast thickness higher than 70 mm), while remaining approximately constant for HE acquisitions (1.18 mGy for less than 30 mm breast thickness, 1.44 mGy for breast thickness higher than 70 mm). The average per-patient MGD for a bilateral two-view CEM was 4.90 mGy. Examining the radiation dose contribution of each CEM component (as normalized the stacked column plot in Figure 6b, providing the relative contribution of the LE and HE images), it can be noticed that the percentage of the total dose attributable to LE images ranged between 64% and 77%, while only the remaining 23–36% was associated with HE images.

## 4. Discussion

The aim of this study was to compare the CEM radiation dose between two prospective studies using the same type of mammography unit and the same CEM protocol, respectively, focused on screening women at increased risk of breast cancer (Center 1, 228 women) and on the work-up of suspicious findings detected at screening mammography (Center 2, 120 women), also deriving an overall estimation of patient dose associated with CEM.

The preliminary physical comparison between the two CEM units showed that the AEC of the unit installed at Center 2 worked with systematically higher EDs compared to Center 1 (mean ED difference was 4.8% for LE images and 5.5% for HE images) to compensate for a slightly lower efficiency and higher noise of the image detector (Figure 2). The similarity of CNR values produced by the two CEM units (mean CNR relative difference: 0.7% for LE images and 0.3% for HE images) indicates that the AECs of the two CEM equipment successfully compensated for differences between the systems, while producing images with a comparable image quality (CNR being the metric used for image quality).

Comparing the two clinical datasets, the median MGD difference between Center 1 and Center 2 was statistically significant (2.33 vs. 2.46 mGy, *p* < 0.001). The 6.2% relative difference in clinical MGD is consistent with the physical differences between the two systems already discussed, but also includes differences between the two study populations, and differences between breast compression in the two centers.

Study population differences were confirmed already by the significantly different median volumetric breast density (13.2% in Center 1 vs. 7.1% in Center 2, *p* < 0.001). As Center 1 enrolled women at increased risk of breast cancer, they were generally younger and often with denser breasts than women at Center 2 (recalled from a standard 50–69 year screening population). In general, the radiation dose required to achieve appropriate image quality from dense breasts is higher than the dose required for non-dense breasts. On the other hand, the 7.2 mm higher breast thickness at Center 2 (47.2 mm at Center 1 vs. 54.2 at Center 2, *p* < 0.001), together with the difference in breast compression (106 N at Center 1 vs. 54 at Center 2, *p* < 0.001), indicate that radiographers at Center 2 applied less firm breast compression compared to their colleagues at Center 1, resulting in higher breast thicknesses and, consequently, higher doses. We also highlight that—if the two centers had used a more similar compression practice—the dose increase at Center 1 (associated with the higher breast density of imaged women) would probably have equaled the systematic dose increase at Center 2 (attributable to technical differences between CEM equipment). The available literature does not provide any specific recommendation about breast compression during CEM exams: at Center 1, given the use of CEM as a first-level examination, radiographers were recommended to apply firm breast compression in order to optimize breast positioning and to reduce as much as possible artifacts on recombined images. Conversely, at Center 2, CEM was part of a much more complex setting with several examinations being sequentially performed on each woman (ultrasound, supplemental DM views or spot compression, and DBT) and radiographers operated without any specific recommendation about compression, therefore potentially drifting towards generally lower breast compression in order to reduce patient discomfort.

Although the CEM MGD difference between the two centers was statistically significant, the clinically not relevant MGD difference (6.2%) and the substantial overlap between the two dose distributions, depicted in Figure 5, allowed us to consider the overall dose distribution obtained by pooling the two datasets. Such distribution encompasses the technical differences between the systems, the differences between the study populations, and the differences between compression techniques. The pooled dataset was used to calculate CEM dose per-patient, by averaging the total CEM dose (sum of CC and MLO dose) between right and left breasts. On average, a standard two-view bilateral CEM exam was associated with MGD values between 3.67 mGy and 6.16 mGy, increasing with breast thickness. LE images, which are substantially equivalent to standard DM images, require that AEC increases LE-MGD as a function of breast thickness to preserve image quality, as occurs for standard DM [24]—indeed, LE-MGD increased from 2.49 mGy for a thickness lower than 30 mm to 4.72 mGy for a thickness higher than 70 mm, with a relative increase of 90%. On the contrary, as the HE X-ray spectra aimed to maximize the iodine absorption, the radiation dose of HE images was substantially independent of breast thickness, ranging from 1.18 mGy for thicknesses lower than 30 mm to 1.44 mGy for thicknesses higher than 70 mm, with a relative increase of only 22%. Considering the total MGD for a bilateral two-view CEM, on average, 70% of total dose is due to the LE imaging and the remaining 30% is associated with the HE component. As LE images can clinically replace standard DM in CEM [4], the dose increase due to the HE additional images is about 30%.

Our results are consistent with those summarized by Hendrick [25], who reported that the CEM dose is 20–45% higher than that delivered by DM, much lower than the 80% dose increase obtained by the initial CEM studies using the prototype equipment [9,10,11,26]. Moreover, our 30% dose increase by CEM compared to standard DM was very similar to the dose increase reported for DBT [27], which is progressively replacing DM in both the diagnostic and screening settings [28]. In fact, while the radiation dose for two-view DBT is reported to range from 3.7 mGy to about 5 mGy, depending on the DBT manufacturer [25], we found a CEM MGD below 5 mGy for any breast thickness below 6 cm. Of note, CEM MGD per-patient at different breast thicknesses found in this study are lower than those reported in a recent single-center study by Bicchierai et al. [29] on another type of CEM unit.

Dosimetric results based on clinical datasets currently available in the literature comparing radiation dose between DM, CEM, or DBT, are usually collected in a single center and are obtained by equipment provided by a single manufacturer. This means that, because of the technical differences between system designs, and possible differences between populations, the relative dose comparison between modalities cannot be generalized. The added value of this study is that the comparison between two datasets from two centers using the same type of equipment also highlighted the existence of possible technical differences between the same model of CEM equipment, and other differences (in this case, between the study populations and compression force applied in the two centers) that can have a measurable impact on the resulting dose.

The results obtained in this study suggest that, as far as the radiation protection principles are applied, CEM can be used for both screening recalls and screening of specific populations, at the cost of a modest dose increase. In particular, when appraising the risk-to-benefit ratio of CEM implementation, it should be highlighted how the functional information provided by CEM in addition to the morphological information coming from LE images would be particularly beneficial for women at increased risk of breast cancer and for women with dense breasts, as a valid alternative to breast MRI, which is much less accessible and much more expensive and time consuming [7,30]. In the group of women at increased risk, particular attention should be paid to mutation carriers (such as BRCA1/2 or P53), taking into account their potential increased radiosensitivity and radiosusceptibility [31], which suggest further evaluation of the risk-to-benefit ratio, also depending on the local accessibility of MRI.

The chief limitation of this study is the inclusion of CEM exams acquired by units of the same model and manufacturer, although some differences were found due to variability between components and calibrations, as well as between the characteristics of the study populations. It could be assumed that larger differences would be obtained in a multi-vendor approach, including CEM systems by multiple manufacturers with different designs.

## 5. Conclusions

Our study found a 6.2% dose difference between two study populations undergoing CEM by the same equipment model, caused by technical differences between the two units, differences between the study populations, and between breast compression practices at the two centers. Overall, the two-view bilateral CEM yielded an average radiation dose of 4.90 mGy, about 30% higher than that of the low-energy mammograms alone, i.e., of a standard digital mammography. Thereby, radiation dose concern should not be an obstacle for future clinical implementations of CEM.

## Figures and Tables

**Table 1:** Characteristics of the study population, obtained by pooling data from two prospective studies using CEM for different screening applications.

Variables		Center 1	Center 2	*p* Value
Demographics	Number of women	228	120	-
	Women age: mean ± SD	51 ± 9 years	59 ± 10 years	<0.001
Breast cancer risk	High ^a^	172/228 (75.4%)	Data not available	-
	Intermediate ^b^	56/228 (24.6%)	Data not available	-
Breast density	Non-dense ^c^	51/228 (22.4%)	66/120 (55.0%)	<0.001
	Dense ^d^	177/228 (77.6%)	54/120 (45.0%)	<0.001

SD, standard deviation. ^a^ High-risk women = women with lifetime risk above 30% (Tyrer–Cuzick risk model). ^b^ Intermediate-risk women = women with lifetime risk between 17% and 30% (Tyrer–Cuzick risk model). ^c^ Non-dense breasts = BI-RADS *a* and BI-RADS *b*. ^d^ Dense breasts = BI-RADS *c* and BI-RADS *d*.

**Table 2:** Tube output and HVL measurement for the two CEM units installed at Center 1 and Center 2.

X-Ray Beam	Tube Output ^a^ (µGy/mAs)		HVL ^b^ (mmAl)			
	Center 1 (Mean ± SD)	Center 2 (Mean ± SD)	Relative Difference(%)	Center 1 (Mean ± SD)	Center 2 (Mean ± SD)	Relative Difference(%)
Mo/Mo@26kV_p_ (LE)	72.3 ± 0.0	69.7 ± 0.0	3.7	0.34 ± 0.0	0.35 ± 0.0	2.9
Mo/Cu@49kV_p_ (HE)	6.9 ± 0.0	6.6 ± 0.0	4.4	3.38 ± 0.0	3.38 ± 0.0	0.0
Rh/Ag@34kV_p_ (LE)	123.4 ± 0.0	117.8 ± 0.0	4.6	0.54 ± 0.0	0.56 ± 0.0	3.6
Rh/Cu@49kV_p_ (HE)	7.7 ± 0.0	7.4 ± 0.0	4.0	3.31 ± 0.0	3.31 ± 0.0	0.0

HVL, half value layer; LE, low-energy; HE, high-energy; SD, standard deviation. ^a^ Tube output is the air-kerma (measured at known distance from the tube exit) divided by the exposure (mAs) value. The distance between X-ray source and dose sensor was 610 mm. ^b^ Half value layer is the thickness of known material which halves the X-ray beam intensity. The material used in mammography is aluminum.

**Table 3:** Relative difference (absolute difference divided by mean value) between the two centers of ED and contrast-to-noise ratio (CNR) from LE- and HE-images obtained by acquiring CEM images of the PMMA phantom at increasing thickness in the automatic exposure (AEC) mode.

PMMA Thickness (mm)	LE-ED Relative Difference (%)	HE-ED Relative Difference (%)	LE-CNR Relative Difference (%)	HE-CNR Relative Difference (%)
20	4.2	5.9	0.7	0.0
25	5.6	6.7	1.1	1.1
30	5.9	6.4	0.9	0.3
35	5.1	5.5	1.2	0.2
40	4.2	5.5	1.5	0.8
45	5.8	4.8	0.4	0.3
50	4.7	5.1	0.7	0.9
55	5.0	4.9	1.3	1.2
60	4.1	5.1	1.9	0.7
65	3.7	5.0	0.7	0.4
70	4.1	5.1	0.4	0.4
Average	4.8	5.5	0.7	0.3

PMMA, polymethyl methacrylate; ED, entrance dose; LE, low-energy; HE, high-energy; CNR, contrast-to-noise ratio. Methods to calculate ED from output measurements and CNR from phantom images have been described in Section 2.2.

**Table 4:** Comparison between total MGD, LE-MGD, and HE-MGD, and between parameters affecting MGD (ED, breast thickness, breast compression, and breast density) obtained from the two clinical datasets.

Parameter	Center 1	Center 2	Median Difference	*p* Value
	Median (IQR)	Median (IQR)	(95% CI)	
Total MGD (mGy)	2.33 (2.19–2.51)	2.46 (2.32–2.70)	0.15 (0.13–0.19)	<0.001
LE-MGD (mGy)	1.52 (1.39–1.73)	1.69 (1.54–1.99)	0.18 (0.15–0.21)	<0.001
HE-MGD (mGy)	0.79 (0.75–0.82)	0.75 (0.70–0.79)	−0.03 (−0.04–−0.02)	<0.001
LE-ED (mGy)	4.37 (3.60–5.68)	5.18 (4.24–7.01)	0.78 (0.60–0.97)	<0.001
HE ED (mGy)	0.86 (0.83–0.90)	0.93 (0.90–0.98)	0.07 (0.066–0.080)	<0.001
Breast thickness (mm)	47.2 (37.5–57.6)	54.2 (45.8–64.2)	7.2 (5.6–8.8)	<0.001
Compression force (N)	106 (90–122)	54 (40–75)	−49 (−51–−46)	<0.001
VBD (%)	13.2 (7.8–20.3)	7.1 (4.4–11.6)	−5.2 (−3.0–−4.4)	<0.001

MGD, mean glandular dose; ED, entrance dose; LE, low-energy; HE, high-energy; IQR, interquartile range; VBD, volumetric breast density. Differences between the two independent samples were tested with the Mann–Whitney U test. *p* values lower than 0.05 were considered statistically significant.

## Data Availability

The datasets used and/or analyzed during the current study are available from the corresponding author upon reasonable request.
